# Supplementary material for: Does small talk with a medical provider affect ChatGPT’s medical counsel? Performance of ChatGPT on USMLE with and without distractions
Source: PLoS One. 2024 Apr 30;19(4):e0302217. doi: 10.1371/journal.pone.0302217 (PMC11060598; doi:10.1371/journal.pone.0302217)
Supplement: Attachment — Submitted filename: Review Small Talk Medical Advice ChatGPT.pdf [file pone.0302217.s002.pdf]

Reviewer background: Statistics, AI, cognitive science

Summary of research and overall impression

The main purpose of the study is to assess the ability of GPT-3.5 and GPT-4 to provide medical advice when small talk is added to LLM input. The input to the LLMs includes questions taken from the United States Medical Licensing Examination (USMLE), which reflect clinical situations that physicians may encounter (i.e. symptoms and medical history of a hypothetical patient). The purpose of the questions is to assess knowledge related to diagnoses and clinical management. Therefore, in this study, the authors use the USMLE questions as a way to assess the LLMs' ability to provide accurate medical advice in two conditions: 1) with small talk sentences added to the questions, and 2) without small talk sentences added to the questions. The percentage of correct responses from the LLMs (i.e. diagnoses, suggested treatment) in both conditions is measured, using responses to the questions from physicians as a ground truth. The authors find two main results. First, GPT-4 provides more correct responses overall compared to GPT-3.5, and second, the small talk negatively impacts the correctness of the responses in GPT-3.5 but not in GPT-4. Another interesting finding is that incorrect responses provided by GPT-3.5 contain no references to the small talk, making it more difficult to identify the error.

Strengths: The topic of the article is very interesting and relevant. The article is well-written (aside from a few grammatical errors) and is easy and enjoyable to read. The description of the data is very clear and the results are clearly described.

Weaknesses: The connection between the motivation of the study and the study itself is weak, and the description of the statistical analyses needs to be elaborated. The discussion could also be elaborated.

Overall impression: It is an interesting study with interesting results that are relevant to the application of generative AI in the medical field, however I believe that the motivation and discussion need some significant work, including a discussion of how generative AI potentially should and should not be used in medicine. Should LLMs be used for tedious tasks such as documentation and note-taking in order to enable doctors to have more time to analyze each case and provide medical advice? Or should LLMs provide medical advice themselves, as an additional resource for doctors? I think is important to make such considerations very explicit in the motivation and discussion in order to justify the value of these results.

Specific areas of improvement

Motivation:

It seems to me that there is a significant disconnect between the motivation of the study and the study itself. The motivation discusses the problem that physicians spend lots of time on documentation and taking medical notes, and that LLMs can help alleviate this burden, but then in the study, medical notes (it seems) are provided as input to LLMs and medical advice is given as outputs. Therefore it is unclear to me how LLMs should arguably be used: for assisting in taking medical notes or for providing medical advice? From my point of view, they are not the same task. Therefore, I think it would be useful in the introduction to specify explicitly what is meant by medical notes and documentation, and to explain how LLMs can aid in these tasks, to avoid this confusion. It is also unclear to me how small talk comes into play. In practice, why would small talk be provided as input to LLMs? I can imagine this being the case if a recording of a conversation between a patient and a doctor is provided to a LLM, but if medical notes

from a doctor are provided to a LLM to aid in a diagnosis, I do not imagine that the doctor would include small talk in their medical notes (though I am not in the medical field). In summary, I think the motivation of the article would be much stronger if these points were made clear explicitly, providing concrete examples of how LLMs can aid physicians, and directly connecting this to the research question of the study.

Discussion:

Similarly, I think the discussion is somewhat vague. The results of the study, which are interesting, would be more valuable if the authors were more explicit about how these results can be put into practice, regarding specifically how generative AI should be/could be used in the medical field.

Statistical analyses:

For the purpose of reproducibility, I think it is important to elaborate on the description of the statistical analyses that were conducted. The Chi-square test of independence was used, but on which variables/groups? Correct vs. Incorrect and Small Talk vs. No Small Talk? What about the difference between GPT-3.5 and GPT-4? I think the authors need to describe how the tests were conducted in more detail.
