# Supplementary material for: Does small talk with a medical provider affect ChatGPT’s medical counsel? Performance of ChatGPT on USMLE with and without distractions
Source: PLoS One. 2024 Apr 30;19(4):e0302217. doi: 10.1371/journal.pone.0302217 (PMC11060598; doi:10.1371/journal.pone.0302217)
Supplement: Attachment — Submitted filename: cover-letter-resp 1.docx [file pone.0302217.s003.docx]

March 12, 2022

Editor-in-Chief

Re: PONE-D-23-29585R1

Dear Editor,

We are pleased to submit our revised manuscript entitled: " Performance of ChatGPT-3.5 and GPT-4 on the United States Medical Licensing Examination With and Without Distractions" for further consideration for publication in Fertility and Sterility.

Thank you for the careful review of our manuscript.

We have addressed the reviewer's helpful comments and are now submitting a revised manuscript. We are grateful for the remarks and suggestions and feel that the current manuscript, incorporating the revisions in response to the comments, is indeed improved. Please find below our point-by-point responses to the reviewers' comments. We appreciate the opportunity to revise our manuscript and hope it will be suitable for publication.

Sincerely,

Myriam Safrai MD

Amos Azaria PhD

Reviewers' comments:

**1 - Reviewer: "***It seems to me that there is a significant disconnect between the motivation of the study and the study itself. The motivation discusses the problem that physicians spend lots of time on documentation and taking medical notes, and that LLMs can help alleviate this burden, but then in the study, medical notes (it seems) are provided as input to LLMs and medical advice is given as outputs. Therefore it is unclear to me how LLMs should arguably be used: for assisting in taking medical notes or for providing medical advice? From my point of view, they are not the same task. Therefore, I think it would be useful in the introduction to specify explicitly what is meant by medical notes and documentation, and to explain how LLMs can aid in these tasks, to avoid this confusion. It is also unclear to me how small talk comes into play. In practice, why would small talk be provided as input to LLMs? I can imagine this being the case if a recording of a conversation between a patient and a doctor is provided to a LLM, but if medical notes* *from a doctor are provided to a LLM to aid in a diagnosis, I do not imagine that the doctor would include small talk in their medical notes (though I am not in the medical field). In summary, I think the motivation of the article would be much stronger if these points were made clear explicitly, providing concrete examples of how LLMs can aid physicians, and directly connecting this to the research question of the study.* ".

**Our response:** Thank you for your feedback on the clarity of our study motivation. First, in the introduction, we have clarified how LLMs can be used for assisting in taking medical notes and how small talks are relevant in the setup between practitioner and patients: "LLMs have the potential to streamline the note-taking process for health practitioners by automating it in two steps. Current technologies enable converting audio information, such as conversations, into transcripts [31]. LLMs can then analyze and create medical notes, such as discharge summaries, in real-time [19]. However, the unique nature of discussions between healthcare providers and patients, which often interweave small talk with critical information, poses a challenge. Given that LLMs are predictive models that generate responses based on the input prompt [35], further investigation is needed to assess whether small talk impedes the LLM's ability to formulate accurate medical notes and summaries."

In addition, at the end of the introduction, we have detailed the application of this study, explaining how the use of LLMs can save time and resources by automating the note-taking process of practitioners and how small talks can interfere with this process in: "To clearly delineate the practical application of this paper, the study targets the use of LLM's alongside a practitioner for charting and summarizing health care patient interaction, including small talk, but with practitioners having significantly less manual work to accomplish. LLM's can save time and resources by automating the note-taking process of practitioners. This requires the ability of the LLM's to process relevant information. However, investigation is required to determine whether small talk inhibits the LLM in formulating accurate medical conclusions. Moreover, LLM's can suggest medical conclusions such as potential treatment and diagnosis, saving time for the practitioner. If small talk interferes with the performance of LLM's, practitioners cannot rely on them for note-taking due to 'noise' causing inaccuracies in medical records."

**2 - Reviewer:** "*Similarly, I think the discussion is somewhat vague. The results of the study, which are interesting, would be more valuable if the authors were more explicit about how these results can be put into practice, regarding specifically how generative AI should be/could be used in the medical field.*

**Our response:** Thank you for highlighting this issue to clarify how these results can be implemented and how and with which caution AI should be used. We added two sentences to the discussion section: " Therefore, according to these findings, the study suggests that ChatGPT3.5 should not be utilized by practitioners for charting and summarizing health care patient interaction. Additionally, our findings suggests that LLM’s have the capacity to streamline the note-taking process for health practitioners to enable doctors to have more time to assess each case and provide medical advice. However, it is important to note that our result also reflected the errors made by ChatGPT in both versions. Therefore, a health practitioner should always be involved as ChatGPT cannot be used independently." A second section summarizing the result and application was added toward the end of the introduction part: " The results demonstrate that for some LLMs (i.e., ChatGPT-4 in our case) adding casual conversation does not impair medical advice or diagnosis made by them. Therefore, some LLMs can potentially be used to generate clinical notes from a written transcript. Today's technology has already automated the conversion of audio transcription to written transcript in real-time. Combining these technologies can reduce the time healthcare workers invest in generating medical notes. However, LLM developers, and especially healthcare providers, must be aware of the limitations present in other LLMs (ChatGPT-3.5 in our case), that do not perform well once clinical information is mixed with casual conversation."

**3- Reviewer:** For the purpose of reproducibility, I think it is important to elaborate on the description of the statistical analyses that were conducted. The Chi-square test of independence was used, but on which variables/groups? Correct vs. Incorrect and Small Talk vs. No Small Talk? What about the difference between GPT-3.5 and GPT-4? I think the authors need to describe how the tests were conducted in more detail.

**Our response:** Thank you for the comment. We rewrote the statistical analysis section and included a detailed description of our analysis: "The study investigates the impact of small talk mixed with medical data on the accuracy of medical advice provided by ChatGPT, comparing its performance between versions 3.5 and 4. The primary outcome measures were the accuracy of responses to USMLE Step 3 questions, both multiple-choice and open-ended, with and without small talk sentences included.

The study employed a mixed-model design, incorporating both within-subject and between-subject factors. The primary independent variable was the presence or absence of small talk sentences added to the USMLE Step 3 questions, while the version of ChatGPT (3.5 vs. 4) served as a between-subject factor.

For each question type (multiple-choice and open-ended), the accuracy of ChatGPT's responses with and without small talk sentences was compared using statistical tests appropriate for the data distribution and study design. Specifically, paired t-tests or Wilcoxon signed-rank tests were conducted to assess within-subject differences in accuracy between conditions (with vs. without small talk sentences) for each ChatGPT version. Additionally, independent t-tests, or Mann-Whitney U tests were used to compare the accuracy of ChatGPT-3.5 and ChatGPT-4 across conditions. Descriptive statistics, including means, standard deviations, medians, and interquartile ranges, were reported to summarize the accuracy of ChatGPT's responses in each condition and for each version. Inferential statistics, such as p-values, were provided to determine the significance of differences observed.

The statistical analyses were performed using Python (version 3.10.1) with the Scipy library (version 1.10.1). The chi2_contingency function from the scipy.Stats library was utilized to compare different groups, and P values of less than 0.05 were considered statistically significant."
